# Biological Activity of Natural and Synthetic Peptides as Anticancer Agents

**DOI:** 10.3390/ijms25137264

**Published:** 2024-07-01

**Authors:** Luana Vittoria Bauso, Valeria La Fauci, Serena Munaò, Desirèe Bonfiglio, Alessandra Armeli, Noemi Maimone, Clelia Longo, Giovanna Calabrese

**Affiliations:** Department of Chemical, Biological, Pharmaceutical and Environmental Sciences, University of Messina, Viale Ferdinando Stagno d’Alcontres, 31, 98168 Messina, Italy; luanavittoria.bauso@unime.it (L.V.B.); valeria_lafauci@outlook.it (V.L.F.); serena.munao@studenti.unime.it (S.M.); desiree.bonfiglio@studenti.unime.it (D.B.); alessandra.armeli@studenti.unime.it (A.A.); noemi.maimone@studenti.unime.it (N.M.); clelia.longo@studenti.unime.it (C.L.)

**Keywords:** cancer, synthetic peptides, small peptides, cancer treatment

## Abstract

Cancer is one of the leading causes of morbidity and death worldwide, making it a serious global health concern. Chemotherapy, radiotherapy, and surgical treatment are the most used conventional therapeutic approaches, although they show several side effects that limit their effectiveness. For these reasons, the discovery of new effective alternative therapies still represents an enormous challenge for the treatment of tumour diseases. Recently, anticancer peptides (ACPs) have gained attention for cancer diagnosis and treatment. ACPs are small bioactive molecules which selectively induce cancer cell death through a variety of mechanisms such as apoptosis, membrane disruption, DNA damage, immunomodulation, as well as inhibition of angiogenesis, cell survival, and proliferation pathways. ACPs can also be employed for the targeted delivery of drugs into cancer cells. With over 1000 clinical trials using ACPs, their potential for application in cancer therapy seems promising. Peptides can also be utilized in conjunction with imaging agents and molecular imaging methods, such as MRI, PET, CT, and NIR, improving the detection and the classification of cancer, and monitoring the treatment response. In this review we will provide an overview of the biological activity of some natural and synthetic peptides for the treatment of the most common and malignant tumours affecting people around the world.

## 1. Introduction

Cancer is a potentially fatal disease due to serious molecular or genomic alterations that lead cells to grow and proliferate in an uncontrolled manner, invading normal tissues and organs and finally spreading throughout the body [1]. With an estimated 20 million new cases and 9.7 million deaths in 2022, cancer is one of the major causes of morbidity and death worldwide. In particular, lung cancer is currently the most frequent malignancy, contributing to 12.4% of all cancer cases globally, followed by breast (11.6%), colorectum (9.6%), prostate (7.3%), and stomach (4.9%) cancers [2].

At present, the most common cancer treatment methods are surgical resection, irradiation, and chemotherapy, adopted alone or in combination [3]. However, these methods show several limits in their effectiveness. Surgery treatment, consisting of complete tumor removal, is an effective approach only in the early stages or when the cancer is still localized, but it is not the best choice in patients with advanced tumours [4]. Radiotherapy and chemotherapy, often used in combination with surgery, are two expensive and long-lasting non-specific approaches that inevitably also damage healthy cells, leading to a variety of side effects [5]. Furthermore, long-term use of chemoteraphy induces chemoresistance, which consists of theinnate and/or acquired capability of cancer cells to evade the effects of chemotherapeutics, making the treatment ineffective and increasing the possibility of recurrence [6]. Consequently, there is an ever-increasing need to test and develop new therapeutic approaches for cancer treatment to overcome the limitations of conventional methods. In the latest years, several molecular biology research studies have led to the discovery of a large number of short peptides with different properties, including antibacterial, antifungal, antitumoral, and even immunomodulatory activities.

Anti-cancer peptides (ACPs) have become a prominent therapeutic approach for cancer treatment due to their great specificity, lower toxicity, and rare side effects [7,8,9]. ACPs are small bioactive molecules ranging in size from 10 to 100 amino acids [10,11], and their source can be natural or synthetic. Natural peptides can be isolated from food, marine products, venom components, and other animal constituents [12,13]; synthetic peptides can be chemically synthesized or obtained through enzymatic hydrolysis and recombinant DNA technology of natural peptides [14]. The majority of ACPs have an amphipathic structure, are positively charged, and show natural antimicrobial properties [15].

**Figure 1:**
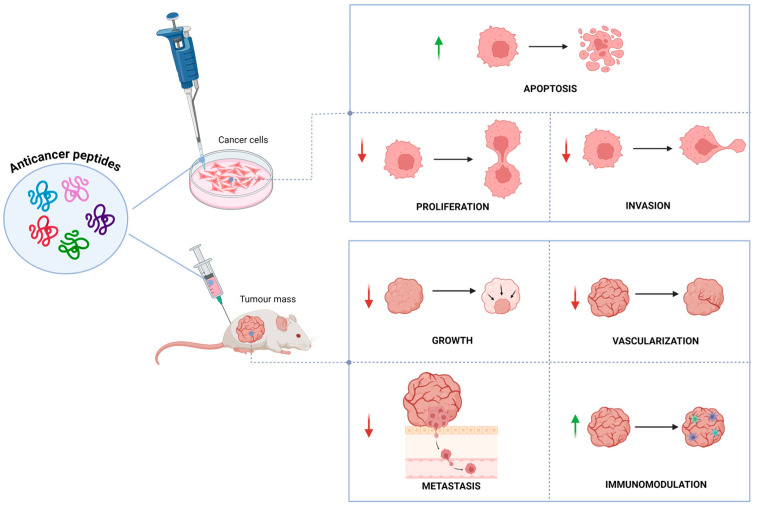
Biological effects of anticancer peptides in vitro and in vivo. Green arrows indicate activation; red arrows indicate inhibition.

They can also have several structural configurations, including both linear and secondary structures like α-helices and β-sheets [16]. ACPs operate at different stages of tumor growth such as initiation, promotion, and progression and specifically target tumor cells. They have remarkable potential over chemotherapeutic drugs, due to their high specificity, greater tumor penetration and fewer side effects. The anticancer activity is presumably due to their interactions with the negatively charged lipids localized on cell membranes, which are found in higher concentrations in cancer cells than healthy cells, leading to a selective death of cancer cells [17]. Various mechanisms for their anticancer property have been documented, including apoptosis induction [18,19], membrane disruption [20], DNA damage [21], angiogenesis inhibition [22], immunomodulation [23], and modulation of pathways involved in cell survival and proliferation [24]. Due to their multiple mechanism of action (Figure 1), biocompatibility, efficient therapeutic efficacy, high tissue penetration, low risk of drug resistance, limited toxicity, and low cost of production, ACPs represent a promising alternative treatment to conventional chemotherapy [25,26].

Furthermore, different ACPs can be used for tumor-targeted delivery of anticancer and natural drugs [27] and for molecular imaging, which are essential for the management of anticancer therapies [28,29,30,31,32,33,34]. Currently, several natural and synthetic peptides are undergoing clinical trials as anticancer therapies. In the Drug Bank Database, 29 peptide or polypeptide-based anti-cancer drugs have been reported. Among these, 5 peptides (Tebentafusp, Buserelin, Plitidepsin, Triptorelin, and Dactinomycin) have been approved for therapeutic purposes by the Food and Drug Administration (FDA) and European Medicines Agencies (EMA), 13 peptides have been studied for anti-tumor therapies (Tigapotide, SF1126, ATN-161, Teverelix, IRL-1620, Nelipepimut-S, Iseganan, G17DT, Canfosfamide, PM02734, CTCE-0214, Darinaparsin, and Labradimil), and 11 peptides (Ozarelix, Soblidotin, LTX-315, Balixafortide, VEGFR2–169, Bombesin, Valspodar, TAK-448, Dolastatin 10, Zoptarelin doxorubicin, and Blemoycin A6) are classified as trial drugs [8].

In light of this evidence, this review aims to provide an overview on the biological effects of novel natural and synthetic peptides for treatment of the six most frequently diagnosed and mortal cancers affecting people worldwide. Furthermore, we also wanted to focus our attention on the role of ACPs on brain and bone tumors which, although show a relatively low incidence, have a mortality rate disproportionately high, specifically among teenagers and young adults. A comprehensive bibliographic search of the last 20 years was carried out using some electronic databases, including PubMed, Scopus, Web of Science, and CancerPPD.

## 2. Lung Cancer

Lung cancer, which develops from lung parenchyma or bronchi, is the primary cause of cancer incidence and death worldwide, accounting for around 2 million cases and 1.8 million deaths (18.4% of all cancer-related deaths) [35].

Lung cancer is a complex disease that may be classified into two types: non-small-cell lung cancer (NSCLC) and small-cell lung cancer (SCLC), which account for 85% and 15% of total lung cancers, respectively. Among NSCLCs, adenocarcinomas are the most prevalent subtypes of lung cancer, followed by squamous-cell carcinomas [36]. Lung cancer is diagnosed routinely through imaging techniques such as chest X-rays, computed tomography (CT), magnetic resonance imaging (MRI), and positron emission tomography (PET) scan; instead, a biopsy (e.g., bronchoscopy, needle biopsy, and surgical biopsy) is frequently required to confirm the diagnosis [37]. Conventional treatment approaches for lung cancer include surgery, radiation therapy, chemotherapy, targeted therapy, and immunotherapy [38]. However, although several therapeutic approaches are available, the prognosis for lung cancer is poor with a low 5-year survival rate [39]. Therefore, it is urgent to provide new approaches for lung cancer treatment. In this context, using peptides could be a promising way to improve the prognosis of patients with lung cancer, given the clinical need to challenge the limits of current therapeutic and diagnosis methods.

Chen et al. [40] evaluated the effects of the MANS peptide, identical to the myristoylated alanine-rich C kinase substrate (MARCKS), significantly expressed in lung cancer, both in vitro and in vivo. Their results demonstrated that the MANS peptide reduces cell migration in vitro and invasiveness of lung cancer cells in vivo, by reducing phosphorylation of MARCKS and AKT/Slug axis.

Park et al. [41] developed a synthetic water-soluble peptide (FCHO1^560–571^), from the protein kinase B (PKB) substrate of human FCH domain only 1 (FCHO1) protein overexpressed in lung cancer. In their in vitro and in vivo studies, they reported that this peptide can inhibit cell proliferation of KRAS-mutated A549 lung cancer cells and significantly suppress tumor growth in A549 xenograft mice by activating PKB/ERK/SMAD4 pathways. In another study [42], the same authors identified another synthetic peptide (TMEM39AS41), derived from the PKB substrate of human transmembrane protein 39A (TMEM39A). Their in vitro results showed that the TMEM39AS41 peptide inhibits cell growth and suppresses inflammation and autophagy pathways, involved in cancer development, in KRAS-mutated cells. Furthermore, they reported that the TMEM39AS41 peptide in KRASLA1 NSCLC mice significantly reduced tumor growth.

Kim et al. [43] synthesized and investigated the effects of a new peptide, MP06, extracted from *Bryopsis plumosa*, a green sea alga. Their in vitro and in vivo results showed that the MP06 peptide can inhibit cell adhesion, migration and invasion, and suppress tumor growth and progression via the ERK signaling pathway.

In another study, Shin et al. [44] designed a new anticancer peptide, AC-P19M, capable of inducing apoptosis, reducing the migration and invasion of lung cancer cells, as well as inhibiting the angiogenic activity of endothelial cells.

Patil et al. [45], in their study, evaluated the activity of a cationic antimicrobial peptide, D-LAK-120A, in vitro and found that it is able to reduce cell proliferation and migration and increase apoptosis of non-small-cell lung cancer. Moreover, they showed that D-LAK-120A suppresses tumor growth and viability in 3D spheroid models of lung cancer.

Jiang and colleagues [46] reported the anti-cancer activity of a peptide-drug conjugate, DTX-P7, consisting of docetaxel (DTX) and a heptapeptide P7 (LPLTPLP), which binds exclusively to the cell surface heat shock protein 90 (Hsp90). They found that the peptide-drug conjugate, preferentially, suppresses tumor growth compared to DTX alone in vivo, with good distribution in tumor tissues. Additionally, they demonstrated that DTX-P7 induces an unfolded protein response leading to cooperative and more effective induction of apoptosis by targeting Hsp90.

## 3. Breast Cancer

Breast cancer is the most frequent tumor and leading cause of cancer-related death in women worldwide. It originates when breast cells undergo genetic alterations that favor their uncontrolled proliferation. This leads to the cells spreading from the breast to other parts of the body [47]. There are various forms of breast cancer that can be defined by the site in which it begins to grow, its growth or spread, and features that influence its behavior. Based on their pathological characteristics and invasiveness, breast cancers can be classified into three groups: in situ or non-invasive (ductal carcinoma in situ), invasive (invasive ductal carcinoma and invasive lobular carcinoma), and metastatic [48]. Further, there are some uncommon types of breast cancers including inflammatory breast cancers, Paget disease, and papillary carcinoma [49,50].

Breast cancer evolves silently and generally is discovered on routine screening by manual or mammography analyses (X-ray) of the breast [51]. Chemotherapy, radiotherapy, and surgical treatment are the main therapeutic approaches for breast cancer. However, the survival rate is still very low due to rapid growth and the incidence of local and distant metastases [52]. Therefore, in the last years, research has been directed towards the development of alternative and more efficacious treatments, including peptide-based therapies [53]. Shi et al. [54] developed a synthetic tumor-specific antigenic peptide with a high affinity for human leukocyte antigen (HLA-A2) (I-6) and evaluated its biological activity against both four cell lines and in a breast cancer murine model. In vitro and in vivo results showed that this peptide significantly induces dendritic cell maturation and promotes the CD8^+^ T immune response, making it a potential candidate for breast cancer immunotherapy. Moreno Ayala et al. [55], in their study, improved the anticancer effect of dendritic cell (DC) vaccines using the Foxp3 blocking peptide P60 in immunocompetent murine models of breast cancer. Their results showed that P60 can inhibit Tregs growth induced by DC vaccines, thus enhancing the therapeutic efficacy of antitumor vaccines. Casanova et al. [56] synthesized linear, dimeric, and tetrameric peptides from lactoferricin B (LfcinB) and evaluated their cytotoxic effect against MDA-MB-468 and MDA-MB-231 breast cancer cell lines. They demonstrated that all LfcinB-derived peptides show a cytotoxic effect against the tested breast cancer cell lines, but tetrameric and dimeric peptides containing the minimal motif (RRWQWR) have a higher cytotoxic potential depending on the concentration of the peptide itself. In another study, Yi et al. [57] reported that synthetic peptides based on Yin Yang 1 protein binding (YPB) and oncoprotein binding (OPB) domain efficiently decrease proliferation and stimulate apoptosis of breast cancer cells, and inhibit tumor growth in vivo, mainly by enhancing the expression of PTENP1 and PTEN and reducing AKT activation.

Li and colleagues [58] synthesized and evaluated the biological effects of a short peptide mimicking human C2ORF40 (C2ORF40MPF) in vitro and in vivo. Their results demonstrated that this peptide significantly reduces breast cancer cell proliferation, migration, and invasion in vitro and tumorigenesis in vivo.

Wu et al. [59] studied the antitumor effect of a natural spider venom-derived peptide, JZTX-14, on the proliferation and migration of MDA-MB-231 breast cancer cells. Their results showed that JZTX-14 does not affect proliferation of breast cancer cells but inhibits their migration by downregulating S100A4 and FBXO2 and upregulating SERPINB2.

## 4. Colorectal Cancer

Colorectal cancer (CRC) is the second most common cause of cancer-related deaths worldwide and the third most frequently diagnosed cancer [60]. CRC develops as an adenocarcinoma; that is, a tumor mass that grows in the cavities of the large intestine and rectum due to the heterogeneity of CRC. CRC arises from serrated sessile lesions or polyps of the colorectal mucosa which, over the years, degenerate and protrude into the lumen of the colon, thus invading the colon wall [61]. It is widely known that 70–80% of CRC cases are sporadic, while 20–30% have a hereditary family history. Factors that may increase the risk of developing CRC include age, unhealthy lifestyle, personal, and family history [62]. Early-stage CRC can often be asymptomatic and lead to poor prognosis. Several diagnostic methods are currently available for CRC, such as physical examination, imaging (abdominal ultrasound, computed tomography scans, and magnetic resonance imaging), colonoscopy or sigmoidoscopy, biopsy, and molecular analysis with specific biomarkers [63]. Treatment options include surgery, chemotherapy, radiotherapy, molecular targeted therapy, and immunotherapy and generally depend on the tumor stage and patient characteristics [3]. Since none of these options are effective in advanced CRC, several efforts are focused on developing new treatments to improve survival rates and quality of life for patients. In this context, a promising new therapeutic approach to conventional treatments is the use of peptides. In their study, Bartolomé et al. [64] tested the synthetic peptide IL13Rα2 D1 showing that it can inhibit proliferation, migration, and invasion in metastatic CRC cells in vitro, and to significantly increase survival in vivo. In another study, Das and colleagues [65] reported that BMAP-27 peptide significantly reduces proliferation and increases apoptosis in colon cancer cells in vitro. Fleten et al. [66] investigated the effects of two oncolytic peptides, DTT-205 and DTT-304, in vivo. Their data demonstrated that intratumoral injection of these peptides leads to complete tumor regression in most treated animals. Hou et al. [67] studied the effects of a peptide-drug conjugate consisting of P-LPK and camptothecin (CPT) (P-LPK-CPT) and found that it can remarkably inhibit tumor growth both in vitro and in vivo by targeting glutamine transporter solute carrier family 1 member 5 (SLC1A5).

## 5. Prostate Cancer

Prostate cancer is the leading tumor affecting males causing a high rate of mortality and morbidity in men worldwide [68]. Prostate cancer can depend on non-variable factors including age, race, and family history, and variable factors such as diet, physical activity, smoking, and obesity [69]. Diagnosis is mainly based on prostate-specific antigen (PSA) screening and transrectal ultrasound-guided (TRUS) prostate tissue biopsies [70,71], but recently new diagnostic approaches have been introduced including PCA3 urine test, Prostate Health Index scoring (PHI), MRI imaging, PIRADS scoring, and MRI-TRUS fusion guided biopsies [72].

There are different types of treatment available for prostate cancer patients, such as surgery, radiation therapy, hormonal treatment, chemotherapy, radiopharmaceutical therapy, immunotherapy, and targeted therapies [73]. Since each of these strategies has different disadvantages, new therapeutic approaches for prostate cancer have been proposed in recent years, including peptide-based treatments.

Nezir et al. [74] developed novel prostate-specific membrane antigen (PSMA)-targeted peptides using phage display technology for the treatment of prostate cancer overexpressing PSMA. They showed that peptide 563 binds PSMA more than peptide 562 thus inducing cell death in vitro. Similarly, Wada et al. [75] investigated the biological activity of a peptide that specifically targets prostate cancer cells, LN1 (C-TGTPARQ-C), showing that it can significantly suppress cell growth both in vitro and in vivo.

In another study, Bossebouf et al. [76] tested the effects of a marine pyroglutamate-modified K092D peptide (pE-K092D) on MDA-Pca-2b prostate cancer cells. They showed that this peptide induces early cytoskeleton perturbation, inhibition of autophagy, inhibition of cell proliferation, and promotes cell necrosis. In other studies, Arap et al. [77] developed a chimeric peptide, consisting of a peptide that binds specifically to the blood vessels in prostate cancer (SMSIARL) and a pro-apoptotic peptide, amphipathic D-amino acid peptide. In vivo results demonstrated that the chimeric peptide slows the tumor development in prostate cancer-prone transgenic mice.

## 6. Gastric Cancer

Globally, gastric cancer (GC) is the fifth most diagnosed cancer and the third leading cause of cancer-related deaths [78]. Several factors may be involved in the development of stomach cancer, including older age, male sex, ethnicity, diet and lifestyle, family history, and genetic predisposition [79,80]. However, pathogenic infections such as *Helicobacter pylori* (*H. pylori*) or Epstein Barr virus (EBV) are responsible for most diagnosed cases of gastric cancer [81,82]. Although the early diagnosis of GC is often delayed, due to its asymptomatic nature, the most used diagnostic methods are esophagogastroduodenoscopy (EGD), endoscopic biopsy, computed tomography scan, endoscopic ultrasound, positron emission tomography scan, magnetic resonance imaging, and chest x-ray [83]. Treatment options for GC include surgery (especially in early stages), chemotherapy, targeted therapy, and immunotherapy [84]. However, despite advances in treatment options, the prognosis of gastric cancer remains poor. Therein, there is the possibility of testing innovative therapeutic strategies, such as peptide-based anticancer therapy. Guo et al. [85] discovered a synthetic peptide, P6-55, capable of significantly inhibiting cell proliferation of gastric cancer cells in vitro and reducing tumor growth in vivo. In another study, Tanaka et al. [86] investigated the effects of a fusion peptide consisting of HIV-TAT and amino acids 331–346 of ephrin-B1 (PTD-EFNB1-C) both in vitro and in vivo. They demonstrated that this peptide suppresses cancer cell invasion by activating RhoA and inhibiting the extracellular secretion of metalloproteinases. Furthermore, they demonstrated that the PTD-EFNB1-C peptide suppresses tumor progression and peritoneal spread in a mouse model of GC. Xing et al. [87] in their study evaluated the anticancer activity of a bioactive peptide-3 (ACBP-3) by targeting miR-338-5p. Their results demonstrated that this peptide inhibits proliferation, induces apoptosis, and reduces the tumorigenicity of human gastric cancer stem cells (GCSCs) both in vitro and in vivo. Furthermore, they reported that ACBP-3 can enhance the therapeutic efficiency of chemotherapy drugs in vivo. In another study [88], the protective effects of an active H-P-6 peptide against *H. pylori*-induced carcinogenesis were investigated. In vitro results showed that the H-P-6 peptide suppresses *H. pylori*-induced hyper-proliferation and migration of gastric epithelial cells. Chen et al. [89] studied the biological activities of a newly identified peptide, GX1, able to selectively target the gastric cancer vasculature. They showed that this peptide inhibits vascular endothelial cell proliferation in vitro and neovascularization in vivo. Further they also evaluated the possibility to conjugate GX1 to rmhTNFalpha, as a targeted delivery system, demonstrating that this system significantly improves the anticancer activity of rmhTNFalpha and decreases systemic toxicity.

## 7. Hepatocellular Carcinoma

Hepatocellular carcinoma (HCC) is the most common primary liver cancer (almost 90%). It ranks sixth among the most frequently diagnosed cancers and is the third leading cause of cancer death [90]. HCC is typically associated with chronic liver disease, particularly in patients with hepatitis B (HBV) or C (HCV) virus [91,92], alcoholic liver disease [93], or non-alcoholic fatty liver disease (NAFLD) [94], occurring in almost 85% of patients diagnosed with cirrhosis. Another risk factor for HCC includes exposure to aflatoxin, a mycotoxin that can contaminate food crops [95]. HCC is more developed in men than women, probably due to the higher frequency of risk factors such as alcohol abuse and chronic HBV and HCV infections [96]. There are currently several therapeutic options for the treatment of HCC, including tumor resection and liver transplantation, radiofrequency ablation (RFA), and chemotherapy [97]. However, although there are several therapeutic options for the treatment of HCC, numerous limitations limit their effectiveness: tumor resection and liver transplantation can be useful for early-stage HCC, but are not suitable in advanced-stage tumors [98]; RFA is effective only for HCC with a diameter less than 3 cm in the early-stage [99]; chemotherapy treatment in advanced HCC is not particularly effective in reducing cancer progression [100]. For all these reasons, new therapeutic approaches for the treatment of HCC have been investigated, including peptide-based therapy [101]. In this regard, Jiang et al. [102] isolated a new peptide from a phage display peptide library, HCC79, capable of specifically binding to hepatoma cell membranes with high affinity. In vitro and in vivo results showed that HCC79 significantly reduces tumor cell migration and enhances tumour inhibition effect by activating T lymphocytes, respectively. Similarly, Zhang et al. [103] identified a synthetic hepatitis C virus core-binding protein 1 (HCBP1) peptide, from a phage display peptide S1 library, able to specifically target HCC cells. In another study, Gusarova et al. [104] investigated the effect of a cell-penetrating alternative reading frame (ARF) peptide, inhibitor of FoxM1, in vitro and in vivo. They found that treatment with an ARF peptide reduces proliferation and induces apoptosis of HCC cells and reduces liver tumor progression in vivo. Sakumaran et al. [105], in a recent work, developed a bioactive peptide (CHP-028) able to target heat shock protein 90 (Hsp90) and cell division cycle 37 (Cdc37) (Hsp90/Cdc37) interaction. They showed that the peptide reduces cell proliferation and increases apoptosis. Recently, Tesauro and colleagues [106] formulated peptide-decorated micelles to deliver highly hydrophobic drugs to HCC cells upregulating the epidermal growth factor receptor (EGFR). They demonstrated that the short peptide D4 (LARLLT sequence) mixed with Pluronic PF127 micelles transports hydrophilic drugs, including doxorubicin, to multidrug-resistant HCC cells overexpressing EGFR.

## 8. Melanoma

Melanoma is the fifth most common cancer in the U.S. and the primary cause of death in patients with skin cancer [107]. Since melanocytes are also found in the urogenital tract, digestive tract, and mucous glands, there are many clinical subtypes of melanoma that differ in demographics, appearance, and molecular profile [108]. The melanoma pathogenesis is multifactorial, mainly due to genetic and environmental factors [109]. Diagnostic approaches for melanoma include physical examination, biopsy (excision or punch), and histological analysis. The primary treatment for melanoma is surgery, which aims to completely remove the cancer; other treatments include radiation therapy, immunotherapy, targeted therapy, and chemotherapy, which depend on the stage of the cancer and overall health. However, despite the advances in therapies for the melanoma treatment, there is still a need to develop novel therapies, particularly for patients with advanced metastatic melanoma. Among the most promising approaches in the last years, anticancer peptides have emerged as a viable alternative to overcome the considerable side effects and poor outcomes of conventional treatments for melanoma [110]. In 2013, Pérez-Torres et al. [111] investigated the therapeutic efficacy of a synthetic parasite-derived peptide, GK1, in a murine melanoma model. Their in vivo studies showed that the GK1 peptide significantly increases the mean survival time and delays tumor growth of the treated animals. Analogously, Camilo et al. [112] evaluated the antitumor activity of LTX-315 against both murine and human cancer cell lines in vitro and a mouse melanoma model in vivo. Their results demonstrated that administration of the LTX-315 peptide induces tumor necrosis and complete tumor regression. Zhao and colleagues [113] evaluated the anti-cancer activity of *Brucea javanica* peptides on melanoma cells. Their results showed that the small molecular peptides inhibit cell proliferation in a dose-dependent manner, arrest the cell cycle, induce cell apoptosis, and inhibit cell migration. In another study, Lee et al. [114] investigated the anti-cancer effect of the insect-derived peptide poecilocorisin-1 (PCC-1) in two melanoma cell lines, SK-MEL-28 and G361. Their in vitro results displayed that PCC-1 reduces cell proliferation, induces apoptosis, and arrests the cell cycle.

## 9. Brain Cancer

Brain cancer is one of the most aggressive forms of cancer with a high mortality rate worldwide. According to the Global Cancer Observatory (GLOBOCAN) 2020 predictions, brain cancer is 12th among the leading causes of tumor-related deaths, and 19th among the most frequent tumors [115]. Primary and metastatic brain tumors are the most common types of brain cancer. Primary brain tumors originate within the brain itself; metastatic brain tumors develop from malignant cells that have spread from other parts of the body [116].

Among primary brain cancers, gliomas are the most prevalent, accounting for approximately 80% of all malignant brain tumors. These tumors originate from glial cells, including astrocytomas, oligodendrogliomas, and glioblastomas. Glioblastoma is known for its aggressive nature and poor prognosis, particularly the more lethal glioblastoma multiforme (GBM), a fast-growing tumor that may result from a lower-grade glioma with frightening global mortality and for which treatment options are low or non-existent. The average survival rate is 15 months [117]. About half of all brain tumors are glioblastomas, whereas 30% are diffusely infiltrating lower-grade gliomas. Meningiomas (2%), ependymomas (3%), and primary central nervous system (CNS) lymphomas (7%) are examples of additional malignant brain tumors [118]. Current diagnostic methods for brain cancers include imaging techniques (magnetic resonance imaging (MRI), computed tomography (CT) scans, positron emission tomography (PET)), and biopsies [119]. The main therapeutic approaches for brain cancers include surgery, radiotherapy, and chemotherapy, which aim to eradicate or reduce the tumor and prevent its spread. However, despite advances in medical technology and treatment options, there are still significant limitations to conventional therapeutic approaches that have led to the investigation of new alternative approaches, such as the peptide-based treatments [120].

In their study, Xin et al. [121] developed a dual targeting drug delivery system for glioma treatment, Paclitaxel (PTX)-loading Angiopep-conjugated PEG-PCL nanoparticles (ANG-PEG-NP-PTX), in order to overcome the blood–brain barrier (BBB) via LRP-mediated endocytosis. Their results showed that ANG-PEG-NP significantly improves the inhibitory effects on the 3D glioma spheroids and the transport across the BBB increasing the penetration, distribution, and accumulation of chemotherapy drugs in glioma.

In another study [26], a targeted delivery system was developed coupling chlorotoxin (CTX), a peptide selective for glioma cells, to liposomes encapsulating antisense oligonucleotides (asOs) or small interfering RNAs (siRNAs) (CTX- SNALPs). In vitro results revealed that coupling of CTX onto the liposomal surface improves particle internalization into glioma cells compared to noncancer cells; nanoparticle-mediated miR-21 silencing increases PTEN and PDCD4 (tumor suppressors), activates caspase 3/7 and reduces the cell proliferation in glioblastoma and glioma cells. Further, in vivo studies showed that CTX improves particle internalization into intracranial tumors. Overall, the results indicate that the development of targeted nanoparticles represents a valuable tool for the targeted delivery of nucleic acid to cancer cells. Wang et al. [122] investigated the potential of Pep-1-conjugated PEGylated nanoparticles loaded with paclitaxel (Pep-NP-PTX) as a targeted drug delivery system for the treatment of glioma via IL-13Rα2 endocytosis. In vitro results demonstrated that compared to NP-PTX, Pep-NP-PTX shows higher uptake and a stronger antiproliferative effect. Further, in vivo results showed a good release of PTX at the glioma site and a significant improvement in the survival of glioma-bearing mice. In another study [123], the authors targeted GBM with a peptide derived from mitochondrial protein voltage-dependent anion channel 1 (VDAC1), crucial for cell energy, metabolism, and apoptosis. They found that the synthetic cell-penetrating VDAC1-based peptide induced cell death in GBM both in vitro and in vivo. In another study, Friedmann-Morvinski and colleagues [124] used a synthetic NEMO-binding domain (NBD) peptide to block the NF-kB pathway, usually upregulated in GBM. They demonstrated that the NBD peptide can reduce tumor proliferation in vitro and prolong survival in vivo. Bidwell et al. [125] investigated the effect of a polypeptide carrier based on elastin-like polypeptide (ELP), modified with a cell penetrating peptide (CPP) and with a therapeutic peptide targeting the oncogenic protein c-Myc (H1). In vitro results showed that the CPP-ELP-H1 peptide strongly inhibits the proliferation of human malignant glioma cells and that its effectiveness was improved by hyperthermia treatment. Further, they reported that CPP-ELP-H1 reduces tumor volume and increases survival time in vivo.

## 10. Osteosarcoma

Osteosarcoma (OS) is a primary bone tumor affecting mostly children and adolescents [126]. It generally originates in the lower limb and the most conventional therapeutic approaches include chemotherapy as well as surgical removal [127]. The chemotherapy is efficient in preventing its progression and recurrence [128]. Nevertheless, when OS metastasizes to the lung, it becomes arduous to treat. Therefore, even today, there is an urgent need to develop innovative and effective treatments to improve the survival of patients with OS. Recently, anticancer peptides (ACPs) have emerged as an encouraging therapeutic approach for several types of cancer, including OS, due to their good penetrability and specificity, and few side effects [129]. Cui et al. [130] evaluated the antitumor capabilities of a new peptide, P05, a fragment of aldolase A (ALDOA), and demonstrated that this peptide shows a strong OS-suppressing capability by inhibiting cell proliferation, motility, and invasion in vitro. In another study, Li et al. [25] used a sarcoma-targeting peptide-decorated disulfide-crosslinked polypeptide nanogel (STP-NG) to deliver a medicinal herb extract, shikonin (SHK), and reduce the progression of OS. Their results reported that STP-NG/SHK can kill OS cells by inducing RIP1- and RIP3-dependent necroptosis. Further, they showed that TP-NG/SHK efficiently reduces tumor growth and pulmonary metastasis in vivo. Yuan et al. [131] investigated the effect of the antimicrobial piscidin 3 peptide (TP3), isolated from Nile tilapia (*Oreochromis niloticus*), on OS cells. Their in vitro results showed that the TP3 peptide significantly inhibits cell viability and increases apoptosis by inducing ROS production and activating caspases 3/9. Kong and colleagues [132] designed a bone-targeting peptide containing eight aspartic acids (D_8_) and an RGD-derived peptide (RGD_2_) conjugated to the surface of gadolinium-doped polydopamine particles for targeted delivery of the nanoparticles to osteosarcoma. They showed that the bone-targeting peptide specifically accumulates at the tumor site and inhibits tumor growth in an osteosarcoma xenograft mice model.

The role of some ACPs on selected cancer types is summarized in Table 1.

## 11. Conclusions and Perspectives

Nowadays, cancer remains a serious public health concern, affecting millions of people annually. Despite advances in cancer treatment, more effective and targeted treatments are still needed, considering the limitations of conventional therapies including surgery, chemotherapy, and radiotherapy. Conventional chemotherapy treatments have several harmful side effects since they are not selective for cancer cells, but also affect healthy cells. Further, cancer cells can develop drug resistance making the chemotherapy inefficacious [133]. Peptide-based therapy, in the last decade, has gained increased interest revolutioning personalized and precision medicine. ACPs are able to act specifically and selectively on tumor cells and do not easily develop drug resistance. In this regard, anticancer peptides are a class of compounds that have shown considerable promise in the detection and treatment of cancer. ACPs therapy affects molecular targets and stimulates biological processes involved in cancer; for example, promoting apoptosis, or preventing growth of cancer cells, as well as triggering antitumor immune responses, or inhibiting tumour irroration, which is essential to provide the high supply of nutrients and oxygen required for the survival of cancer cells [134].

ACPs can be used as targeted delivery systems that allow anticancer drugs to be delivered to specific sites thereby reducing damage to healthy cells and increasing efficacy against tumor cells. Among the tumor-targeting peptides identified thus far, NGR (Asn-Gly-Arg) and RGD (Arg-Gly-Asp), cell-penetrating peptides (TAT peptides), Angiopep-2, and other peptides have proven useful for delivering chemotherapeutic drugs, antiangiogenic agents, apoptotic peptides, cytokines, nucleic acids, and proteins [135]. Moreover, targeting peptides can be combined with imaging agents to selectively target cancer cells, offering more accurate and earlier tumour detection and classification, as well as monitoring response to therapy. For example, different NGR motifs have been labeled with different radionuclide, such as ^68^Ga and ^64^Cu for positron emission tomography (PET) imaging and ^99m^Tc for single-photon emission computed tomography (SPECT) imaging [1].

It is widely recognized that compared to traditional drugs, peptides possess several advantages including low risk of drug resistance, high biocompatibility, high tissue penetration, high selectivity and specificity for targets, low toxicity, easy elimination from the body, increased therapeutic efficacy, and lower capacity for side effects. Nevertheless, the ACPs application for therapeutic purposes has been limited by a variety of issues such as low half-life, low bioavailability, limited intestinal permeability linked to polarity and molecular weight, and susceptibility to enzymatic degradation. Furthermore, the divergence between in vitro and in vivo efficacy prevents clinical translation [136]. Despite these problems, researchers and pharmaceutical companies continue to collaborate to find new valid peptides to translate clinically.

To address these concerns, different modification strategies, innovative design methods, and novel nanotechnology-based delivery systems (i.e., liposomes, nanoparticles, and micelles) have been adopted and have proven to be helpful both in vitro and in vivo. Hence, researchers are evaluating the possibility to use nanosystems due to their prolonged drug half-life, enhanced stability, selectivity, and bioavailability [137]. Furthermore, nanosystems improve the absorption and distribution of drugs, which can bypass enzymatic activity, allowing the exploitation of their great therapeutic potential. Currently, the integration of peptides into artificial materials has become a successful approach to overcome their limits and improve the surface properties of materials for several applications. For example, the development of self-assembled systems with ACPs and chemotherapeutic drugs may represent an attractive strategy for the treatment of cancer [138].

Despite peptide-based nanosystems representing an advantageous approach for personalized therapies, many limitations are still preventing their clinical translation, including their complex effect, standardization, and production. Therefore, further studies are needed to contribute to the successful clinical translation of peptide-based nanosystems. Therefore, it is very likely that peptide-based nanosystems may soon represent the new prophylactic and therapeutic regimen against cancer [139,140,141,142].

The purpose of this study was to illustrate the latest advances in the application of natural and synthetic peptides as anticancer therapeutic agents, providing an overview of their biological activity in some of the most diagnosed and deadly cancers worldwide. The results shown in the studies discussed in this review indicate that ACPs can be considered promising agents for developing new anticancer drugs and vaccines, to reduce the incidence of new cancer cases and the mortality rate.

## Figures and Tables

**Table 1:** Natural and synthetic anticancer peptides and their biolocical activity in vitro and in vivo.

Tumor Type	Peptide Name	Peptide Sequence	Biological Activity	In Vitro/ In Vivo Model	Ref.
**Lung cancer**	MANS	Myrisitic Acid-GAQFSKTAAKGEAAAERPGEAAVA-OH	Reduces cell migration in vitro and invasiveness in vivo	PC9, A549, and NCI-H29 cell lines; mouse	[40]
	FCHO^1560–57^	560PPRRLRSRKVSC571	Inhibits cell proliferation in vitro and tumor growth in vivo	K^RAS^-mutated A549 cell line; mouse	[41]
	TMEM39AS41	34GLRNRNGSAIGLPVP48	Inhibits cell growth and suppresses inflammation in vitro; reduces tumor growth in vivo	A549 cell line; mouse	[42]
	MP06	LAVISWKCQEWNSLWKKRKRKT	Inhibits cell adhesion, migration, and invasion in vitro, and suppresses tumor growth and progression in vivo	A549, H460 and H129 cell lines; zebrafish embryos	[43]
	AC-P19M	FAKKLAKLKKKLAKLAKKR	Induces apoptosis, reduces migration and invasion, inhibits angiogenic activity of endothelial cells	A549, H460 and HUVECs cell lines	[44]
	D-LAK-120A	KKLALALAKKWLALAKKLALALAKK-NH2	Reduces cell proliferation and migration, increase apoptosis of NSLC cells; suppresses tumor growth and viability in 3D spheroid models of lung cancer	A549, H358, H1975, and HCC827 cell lines and 3D spheroid model of NSCLC	[45]
	DTX-P7	LPLTPLP	Inhibits tumor growth and induces apoptosis	A549 cell line; mouse	[46]
**Breast** **cancer**	I-6	SQYGEPRKL	Promotes dendritic cell maturation and CD8^+^ T immune response	MDA-MB-231, MCF-7, MCF-10A cell lines; mouse	[54]
	P60	RDFQSFRKMWPFFAM	Enhances therapeutic efficacy of antitumor vaccines	4T1, LM3, MCF7, MDA-MB-231 cell lines; mouse	[55]
	LfcinB-derived peptide	RRWQWR	Cytotoxic effect in vitro	MDA-MB-468, MDA-MB-231 cell lines	[56]
	YPB OPB	PPRKKKRKHRLWAAHCRKIQLKKDGSS GNKKWEQKQVQIKTLEGEFSVTMWSS	Reduce cell viability in vitro, inhibit tumor growth in vivo	MDA-MB-231; MDA-MB-453; MCF-10A and MCF-7 cell lines; Mouse	[57]
	C2ORF40MPF	SPYGFRHGASVNYDDY	Reduces proliferation, migration, and invasion in vitro; tumorigenesis in vivo	BT549, MDA-MB-23 cell lines; mouse	[58]
	JZTX-14	GCQKFFWTCHPGQPPCCSGLACTWPTEICILGR	Prevents migration and invasion in vitro	MDA-MB-231 cells	[59]
**Colorectal cancer**	IL13Rα2 D1	GSETWKTIITKN	Inhibits proliferation, migration, and invasion in vitro, and increases survival in vivo.	KM12SM cell line; mouse	[64]
	BMAP-27	GRFKRFRKKFKKLFKKLSPVIPLLHL	Reduces proliferation and increases apoptosis in vitro	SW480 and SW620 cell lines	[65]
	DTT-205 and DTT-304	-	Tumor regression	Mouse	[66]
	P-LPK-CPT	LPKTVSSDMSLN-CPT	Inhibits tumor growth both in vitro and in vivo	Colo320HSR, HCT116, LoVo, HT29, and SW480 cell lines; mouse	[67]
**Prostate cancer**	562	N′-SHSFSVGSGDHSPFT-C′CQK	Binds PMSA and induces cell death in vitro	PNT1A, 22Rv1, and LnCaP cell lines	[74]
	563	N′-GRFLTGGTGRLLRIS-C′	Binds PMSA and induces cell death in vitro	PNT1A, 22Rv1, and LnCaP cell lines	[74]
	LN1	C-TGTPARQ-C	Suppress cell growth both in vitro and in vivo	PC3 cell line; mouse	[75]
	pE-K092D	pE-QLTPEALADEEEMNALAAR	Induces cytoskeleton perturbation, inhibits autophagy, inhibits cell proliferation, and promotes cell necrosis	MDA-Pca-2b cell line	[76]
	SMSIARL	_D_(KLAKLAK)_2_	Slows the tumor development in vivo	mouse	[77]
**Gastric** **cancer**	P6-55	RKKRRQRRRLKSAHYIELGSYQYWPVLVPRGIRLYTYEQIPGSLKDNPYITDGYRAYLP	Inhibits cell proliferation in vitro and reduces tumor growth in vivo	AGS and MKN45 cell lines; mouse	[85]
	PTD-EFNB1-C	GRKKRRQRRRPPQGGGVQEMPPQSPANIYYKV	Suppresses cancer cell invasion in vitro and tumor progression and peritoneal spread in vivo	44As3 cell line; mouse	[86]
	ACBP-3	-	Inhibits proliferation, induces apoptosis, and reduces the tumorigenicity in vitro and in vivo.	GC MKN45 cell line;	[87]
	H-P-6	Pro-Gln-Pro-Lys-Val-Leu-Asp-Ser	Suppresses *H. pylori*-induced hyper-proliferation and migration in vitro	AGS enteric epithelial cells	[88]
	GX1	CGNSNPKSC	Inhibits vascular endothelial cell proliferation in vitro and neovascularization in vivo	HUVEC and SGC7901 cell lines; mouse	[89]
**Hepatocellular** **carcinoma**	HCC79	KSLSLHDHHHH	Inhibits tumor invasion	SMMC-7721 cell line; mouse	[102]
	HCBP1	FQHPSFI	Binds specifically to hepatoma cells	HepG2, BEL-7402, L-02 cell lines;	[103]
	ARF_26–44_	KFVRSRRPRTASCALAFVN	Reduces proliferation and induces apoptosis in vitro; reduces liver tumour progression in vivo	PLC/PRF/5, Hep3B, HMEC-1, HepG2 cell lines; mouse	[104]
	CHP-028	CH3-CO-KTGDEK-K-GG-YGRKKRRQRRR-NH2	Inhibits cell proliferation and migration, induces apoptosis	HepG2 and Huh7 cell lines	[105]
	D4	LARLLT	Delivers hydrophobic drugs to cancer cells overexpressing EGFR	HepG2, Hep3B, Huh7, and PLC/PRF/5 cell lines	[106]
**Melanoma**	GK1	GYYYPSDPNTFYAPPYSA	Increases the mean survival time and delays tumor growth in vivo	Mouse	[111]
	LTX-315	KKWWKKW-Dip-K-NH2	Induced tumor necrosis in vitro and tumor regression in vivo	B16F1, MRC-5, HUV-EC-C, and A375 cell lines; mouse	[112]
	*B. javanica*	-	Inhibits cell proliferation, induces cell apoptosis, and inhibits cell migration	A375 cells	[113]
	PCC-1	KKRKKKAFALKFVVDLI-NH2	Reduces cell proliferation and induces cell cycle arrest and apoptosis	SK-MEL-28 and G361 cell lines	[114]
**Brain cancer**	ANG-PEG-NP-PTX	TFFYGGSRGKRN NFKTEEY	Induces cell death and inhibits spheroids growth in vitro; enhances penetration, distribution, and accumulation of chemotherapeutic agent in vivo	Spheroids of U87 MG cell line; mouse	[122]
	CTX- SNALPs	MCMPCFTTDHQMARKCDDCCGGKGRGKCYGPQCLCR	Increases PTEN and PDC4; activates caspase 3/7 and reduces cell proliferation in vitro; enhances particle internalization in vivo	F98 rat and GL261 mouse glioma cell lines; mouse	[26]
	Pep-NP-PTX	ACGEMGWGWVRCGGSLCW	Enhances uptake and reduces cell proliferation in vitro; enhances survival of glioma-bearing mice	C6 cell line; mouse	[122]
	VDAC1-based peptides	Tf-D-LP4, HAIYPRHSWTWE-199-KKLETAVNLAWTAGNSN-216-KWTWK, Retro-Tf-D-LP4, KWTWK-216-NSNGATWALNVATELKK-199-EWTWSHRPYIAH, D-ΔN-Ter-Antp, 15-RDVFTKGYGFGL-26- RQIKIWFQNRRMKWKK	Induce cell death in vitro; inhibit tumor growth in vivo	U-87MG, U-118MG, U-251MG and LN-18, SH-SY5Y, GL-261MG, and Neuro-2a cell lines; mouse	[123]
	NBD	TALDWSWLQTE	Reduces tumor proliferation in vitro; protracts survival in vivo	U-87MG, SK892, SK429, and SK748 cell lines; Mouse	[124]
	CPP-ELP-H1	WPGSGNELKRAFAALRDQI	Inhibits cell proliferation in vitro; reduces tumor volume and increases survival time in vivo	Human U-87-MG and D54 cell lines; rat C6 cell line; rat	[125]
**Osteosarcoma**	P05	ADDGRPFPQVIK	Inhibits cell proliferation, motility, and invasion in vitro	U2OS cell line	[130]
	STP-NG/SHK	STP-PEG-P(LP-co-LC)	Induces necroptosis in vitro; suppresses tumor growth and reduces pulmonary metastasis in vivo	hFOB1.19, human osteosarcoma 143B cell lines; mouse	[25]
	TP3	H-FIHHIIGGLFSVGKHIHSLIHGH-OH	Inhibits cell viability and increases apoptosis	MG63 cell line	[131]
	PDA@Gd-D_8_/RGD_2_	DDDDDDDDC RGDfRGDfC	Inhibits tumor growth in vivo	mouse	[132]

## Data Availability

No new data were created or analysed in this study. Data sharing is not applicable to this article.
